# A MODEL ACCESSORY-DWELLING UNIT BYLAW: INCLUSIVE ZONING FOR ALL

**DOI:** 10.1093/geroni/igad104.2890

**Published:** 2023-12-21

**Authors:** Travis Gagen

**Affiliations:** Springfield College, Springfield, Massachusetts, United States

## Abstract

Adapting the built-environment to include an accessory-dwelling unit (ADU) is one alternative housing solution for older adults to remain in their home and community, avoid institutionalization, and create age-friendly environments. ADUs are one form of the built-environment in which the field of public health law can intervene to improve population aging. A legal mapping analysis was conducted using the ADU Friendliness Score to assess ADU accessibility across the target population (n = 351 Massachusetts municipalities). Municipalities were stratified into four ordinal classifications according to their ranking on the 0-100 ADU Friendliness Score and served as the dependent variable: 0-24 POOR (89 municipalities, 25%); 25-49 FAIR (30 municipalities, 8.5%); 50-74 GOOD (185 municipalities, 53%); and 75-100 EXCELLENT (47 municipalities, 13.5%). Community demographics and social capital proxy measures were used as independent variables to test for the association between aggregate social capital and the dependent variable. A regression analysis demonstrated housing density and educational attainment as statistically significant predictors of ADU score. Findings informed the first model age-friendly ADU bylaw inclusive for older adults, persons with disability, and their caregivers. Reform of zoning laws can help create age-friendly environments. The model bylaw is available for municipals to adopt and can be modified in response to municipal needs.

